# Effect of a conditional cash transference program on food insecurity in Mexican households: 2012–2016

**DOI:** 10.1017/S1368980021003918

**Published:** 2022-04

**Authors:** Mariana Saldivar-Frausto, Mishel Unar-Munguía, Ignacio Méndez-Gómez-Humarán, Sonia Rodríguez-Ramírez, Teresa Shamah-Levy

**Affiliations:** 1 Nutritional and Health Research Center at the National Institute of Public Health, Cuernavaca, Morelos, México; 2 Center for Research in Mathematics, Aguascalientes Unit, Aguascalientes, Mexico; 3 Evaluation and Survey Research Center at the National Institute of Public Health, Universidad No. 655, Colonia Santa María Ahuacatitlán, Cerrada Los Pinos y Caminera, 62100 Cuernavaca, Morelos, México

**Keywords:** Food insecurity, Program effect, Mexican households, Cash transference programs

## Abstract

**Objective:**

To estimate the effects of the social inclusion programme PROSPERA on food insecurity (FI) in Mexican households during 2012 and 2016.

**Design:**

Quasi-experimental study using cross-sectional data from 2012 to 2016 National Household Income and Expenditure Survey – Socioeconomic Conditions Module (in Spanish, ENIGH-MCS).

**Setting:**

Data were used from a 2012 sample of 56 888 Mexican households (representative of 31 206 819 households) and a 2016 sample of 70 263 Mexican households (representative of 33 445 353 households). Severity of FI was estimated with the Mexican Food Security Scale (in Spanish, EMSA). The statistical analysis estimated a differences in differences (DD) model weighted by propensity score to compare program beneficiary and non-beneficiary households in 2012 than in 2016. We estimated the effect on households with and without children (< 18 years of age). We also compared this model to a DD model without propensity score weighting.

**Participants:**

Mexican households.

**Results:**

FI among all beneficiary households decreased 8·0pp as compared to non-beneficiary households over the study period. In beneficiary households with children, this decrease was 6·0pp and for beneficiary households without children, this decrease was 12·9pp (for all, *P*-value < 0·001).

**Conclusions:**

The PROSPERA program had a positive effect on FI reduction at the household level through increasing food access, which usually improves nutritional outcomes in vulnerable Mexican populations.

Food insecurity (FI) is ‘the limited or uncertain availability of safe and nutritionally adequate food, or uncertainty in the ability to acquire adequate food in socially acceptable ways’^(1)^. Scales which measure FI are often based on experiences^(2)^, where populations report whether or not they feel they have the resources to ensure adequate food quantity, quality and diversity in their diet. Therefore, FI is an indicator of food access^(3)^.

Each level of severity of FI implies a different stage of food deprivation; the mild level corresponds with a reduction in dietary variety and quality, a moderate level means a reduction in the quantity of food consumed by adults and the severe level is associated with a complete lack of food in the household and/or that food has been obtained only by socially unacceptable means such as begging or child labour^(4)^.

FI continues to be a major problem in Mexico, as revealed by the Encuesta Nacional de Salud y Nutrición (ENSANUT), which in 2012 reported that seven of every ten households reported some level of FI: 41·6 % mild FI, 17·7 % moderate and 10·5 % severe. According to ENSANUT 2012, the total proportion of Mexican households experiencing food deficiency (defined as the combined prevalence of moderate and severe FI) was 28·3 %^(5)^, while in the same year the Consejo Nacional de Evaluación (CONEVAL) reported 23·3 %^(1)^. In 2016, the ENSANUT-MC found that 69·5 % of Mexican households experienced some level of FI, (40·1 % mild, 18·4 % moderate and 11·1 % severe)^(6)^, whereas CONEVAL reported that 20·1 % of households experienced food deficiency^(7)^.

Worldwide, many programs have been launched with the goal to minimise household vulnerability and interrupt the intergenerational poverty cycle, using different strategies such as the formation of human capital^(8)^, or offering subsidies or cash to low-income families to improve nutrition status, education and health of specific population groups^(3)^. These monetary incentives – also called conditional cash transfer (CCT) programs^(9)^ – are an example of interventions which employ a combination of actions aimed at addressing the immediate and underlying determinants of conditions such as malnutrition^(8)^.

CCT programs can impact health and nutrition outcomes through monetary incentives, which increase the buying power of beneficiary households. However, they also provide other services, such as nutrition and health education which are meant to condition the preferences and attitudes of families, basic health services either as part of the program or through complementary strategies for healthcare expansion in program areas, or through preferential or facilitated access to services^(10)^.

CCT programs have been implemented in Mexico, Brazil and Bangladesh since 1997^(11)^, and have spread rapidly such that by 2016, sixty-three low- or middle-income countries were implementing at least one CCT^(12)^. Today, there are active CCT programs on every continent, in both high- and low-income countries^(11)^.

In Mexico, the program for education, health and food (PROGRESA) initially served 300 000 beneficiary households, all in rural areas^(13)^; subsequently (in early 2002), PROGRESA served 2·4 million households, of which two-thirds were in indigenous communities^(14)^ and new urban households^(13)^. In mid-2002, the program was modified and renamed ‘Programa de Desarrollo Humano – Oportunidades’ (hereafter, Oportunidades), simultaneously increasing its coverage to 4·2 million households across all 32 Mexican states^(14)^.

By 2014, the National System for the Crusade Against Hunger was established^(15)^, and Oportunidades was modified to become the ‘PROSPERA Social Inclusion Program’ (hereafter, PROSPERA)^(14)^. PROSPERA allocated additional cash towards achieving nutritional gains in beneficiary households, including eliminating acute child malnutrition and improving child weight and height indicators. It also aimed to: increase food production and income of farmers and small growers; minimise post-harvest food losses during storage, transportation, distribution and marketing and promote community participation towards eradicating hunger^(16)^. By 2016, this program served over six million households throughout Mexico^(17)^.

The effects of this CCT (PROGRESA-Oportunidades-PROSPERA) on the health of beneficiary children and their mothers have been widely described, and include outcomes such as increased height, higher haemoglobin levels^(18)^, decreased prevalence of anaemia^(19)^, decreased morbidity^(20)^ and reduced low birthweight^(21)^. Notably, the nutritional outcomes evaluated in PROSPERA over time have focused on individual-level evaluation, using malnutrition indicators focused only on children^(22)^, women^(23)^ and/or pregnant women^(24)^. Although the program was not designed to reduce FI, nutrition improvements reported in beneficiaries may be derived from greater access to food^(25)^, as previously observed in Brazil^(26)^. Therefore, in this study, we aimed to analyse the effect that PROSPERA may have had on household-level FI. It should be noted that, in 2019, the PROSPERA program was terminated and its budget was reassigned to create the National Liaison for Wellbeing Scholarships - Benito Juárez^(27)^. Nevertheless, this program with its broad trajectory and wide coverage throughout the Mexican population, and which demonstrated positive results across multiple areas (health, education and nutrition) justifies the analysis of its other possible outcomes, such as FI. This approach bolsters program evaluation in Mexico, as it reveals another method of studying interventions and their scope, and provides evidence on the effects of CCT programs on beneficiary households.

## Methods

**Fig. 1:**
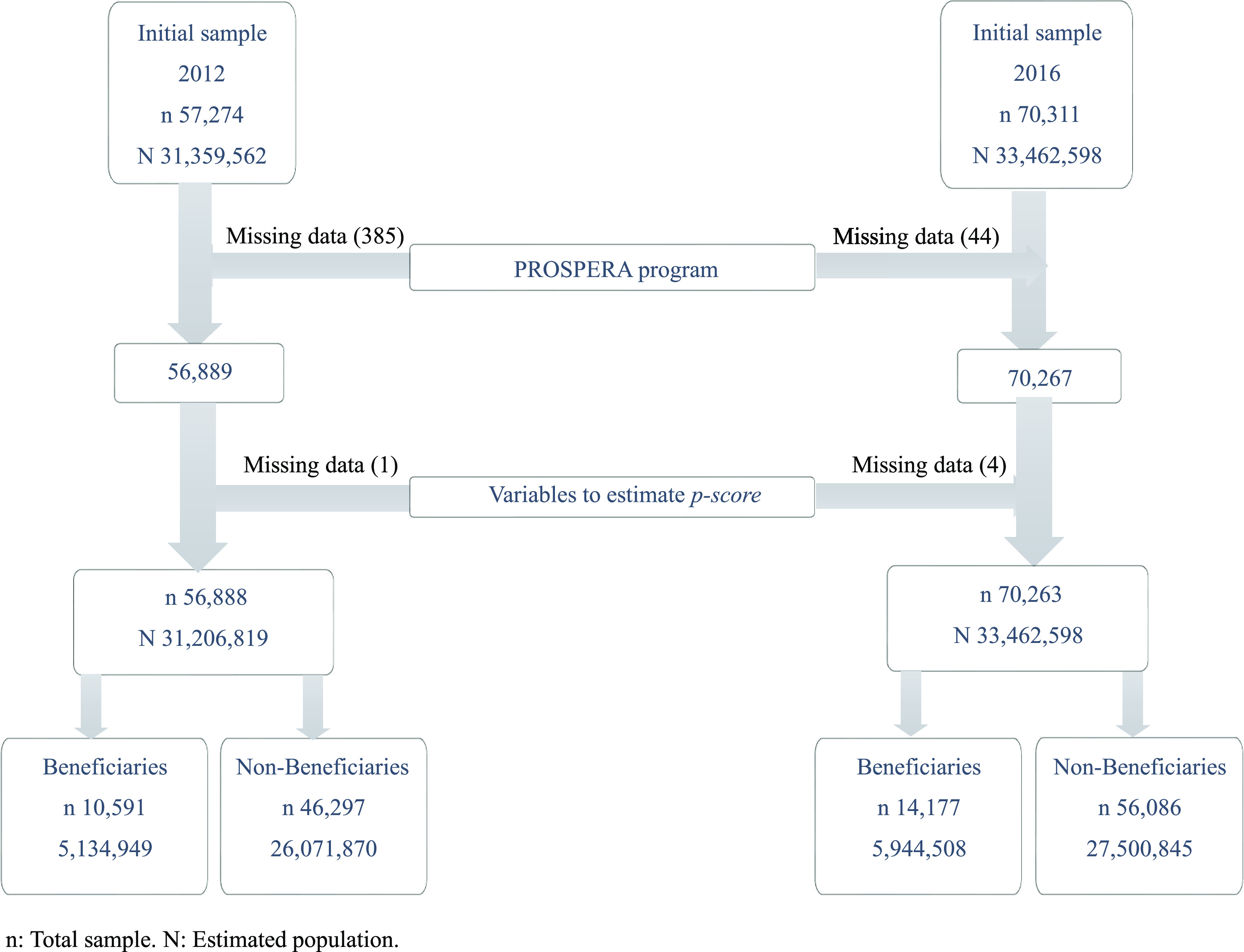
Analytical sample

We performed an observational comparative study with two groups, based on secondary individual- and household-level data from the years 2012 and 2016 from a cross-sectional survey known as the National Household Income and Expenditure Survey – Socioeconomic Conditions Module (in Spanish, ENIGH-MCS). This survey is characterised by a probabilistic design allowing extrapolation to the entire Mexican population, and has been discussed in detail elsewhere^(28,29)^. Our analysis was performed with the sample described in Fig. 1.

Beneficiary households of the PROSPERA program received cash transfers on the condition that children remained enrolled in school and the household members received healthcare services, including nutritional orientation^(30)^. For our study, PROSPERA beneficiary households were considered to be those which reported at least one family member receiving program benefits (as described above) within the last month; otherwise, they were classified as non-beneficiary households.

### Household FI

We analysed food access to determine the severity level of FI as measured by the Mexican Food Security Scale (in Spanish, EMSA) in PROSPERA beneficiary households in 2012 and 2016, using the CONEVAL methodology for multidimensional poverty measurement^(2,31)^. The EMSA is based on self-reported experiences relating to household food access, and is described in detail in the Supplementary Material^(32)^. The scale includes 12 questions listed in order from least severe to most severe, and examines whether household members have experienced a decrease in food variety, quantity or quality, as well as episodes of hunger in the last 3 months due to lack of income or other resources^(32)^.

To classify severity levels of household FI, we added all affirmative responses considering the household composition (presence of adults and children). Those households with no affirmative responses were classified as food secure. For households with only adults, the maximum score possible was 6; 1–2 affirmative responses signified mild FI; 3–4 affirmative responses signified moderate FI and 5–6 affirmative responses signified severe FI. In the case of households with members under 18 years of age, the maximum score possible was 12; 1–3 affirmative responses signified mild FI; 4–7 affirmative responses signified moderate FI and 8–12 affirmative responses signified severe FI.^(33)^

For purposes of this analysis, the FI variable was also dichotomised; all severity levels (mild, moderate and severe) were aggregated in one category which indicated overall FI (FI = 1), and a reference category for those categorised as food secure (FI = 0).

Variables that are used to estimate multidimensional poverty in Mexico^(33)^ also characterise the sample at the family, household, local and regional levels. These are described below:

### Household member characteristics

#### Head-of-household

We constructed three variables to describe the head-of-household: sex (binary); average age (tertile) and education level (categories). The age tertiles (T) for the 2012 survey were: T1 (12–40 years), T2 (41–55 years) and T3 (56–97 years), while for 2016 the tertiles were: T1 (14–40 years), T2 (41–55 years) and T3 (56–105 years). Education level was defined as the maximum grade of formal studies undertaken, and was categorised into four categories: (1) none or kindergarten, (2) elementary school (complete or incomplete), (3) middle school (complete or incomplete) and (4) high school or above.

#### Indigenous background

This variable is considered affirmative when any household member over 3 years of age speaks an indigenous language.

#### Household size

This is a numeric variable based on the adult equivalency scale (of economies of scale) which describes household composition by assigning values by a member according to age range. A household member with 19 years or more counts as 0·9945, 18–13 years as 0·7057, 12–6 years as 0·7382 and 5–0 years as 0·7031^(31)^. These values are multiplied by the number of members of each age range, then added together.

#### Proportion of women

This variable represents the proportion of female household members in relation to total household members.

### Household characteristics

#### Lack of healthcare access

We considered lack of healthcare access to be the lack of affiliation to any public or private institution providing medical services, either through employment benefits, voluntary recruitment or direct family affiliation through a relative; otherwise, households were classified as not lacking healthcare access.

#### Economic vulnerability

In this binary variable, marginalised households (or, those with unmet basic needs) and whose monthly income was below the poverty line for each year were considered as economically vulnerable. In both 2012 and 2016, the poverty line was defined as a monthly income of $2329 MXN (approx. $116 USD) for urban households and $1490 MXN (approx. $79 USD) for rural households^(34,35)^. If households did not have either characteristic, they were considered not economically vulnerable.

#### Lack of quality housing materials

This variable was considered to be affirmative for households with dirt floors, or ceilings and walls built with waste materials or cardboard; otherwise, households were classified as not lacking quality housing materials.

#### Overcrowding

This variable considers the total of individuals within the household divided by the total rooms. Households were classified as overcrowded if this value was greater than 2·5.

#### Basic public services

These binary variables considered whether or not households lacked access to the following services (detailed description published elsewhere)^(33)^: water, sewage, electricity and gas.

#### Marginalisation index

A marginalisation index was constructed from indicators (dichotomous variables) describing dimensions of ineffective social access, including the lack of access to: education, healthcare, social security, quality housing and public spaces, basic public services and food. The criteria and variables which make up these indicators have been described in detail by CONEVAL^(33)^.

Using these data, we created a simple summary index representing the number of social access dimensions not being satisfied in households. We grouped households who reported affirmative responses in five or six dimensions into a single category, such that the marginalisation index consisted of categories from zero to five.

### Local and regional characteristics

#### Locality type

We classified household localities as rural when they had less than or equal to 2500 inhabitants; localities with over 2500 inhabitants were classified as urban.

#### Region

We considered four geographical regions in Mexico: (1) North as Baja California, Baja California Sur, Coahuila, Chihuahua, Durango, Nuevo León, Sonora, Sinaloa, San Luis Potosí, Tamaulipas and Zacatecas; (2) Center as Aguascalientes, Colima, Guanajuato, Hidalgo, Jalisco, Michoacán, Morelos, Nayarit, Querétaro and municipalities of the State of Mexico not in the metropolitan area; (3) Mexico City as the Federal District and metropolitan municipalities of the State of Mexico and (4) South as Campeche, Chiapas, Guerrero, Oaxaca, Puebla, Tlaxcala, Quintana Roo, Tabasco, Veracruz and Yucatán.

### Statistical analysis

We performed a descriptive analysis on household characteristics for 2012 (time point 0) and 2016 (time point 1), as well as on the prevalence of each level of FI severity. We used regression analysis to estimate and test whether the proportions and averages between the years 2012 and 2016 were statistically different.

To assess the effect of time in the PROSPERA program on FI, a differences in differences (DD) model was performed through a logistic regression and was weighted by propensity score. To improve internal validity, the propensity score was used to estimate the probability of being a beneficiary household and a control group of non-beneficiary households was selected. The variables used to determine the propensity score were: (1) sociodemographic characteristics of household members: head-of-household, indigenous background, household size, proportion of women; (2) household characteristics: lack of healthcare access, economic vulnerability, lack of quality housing materials, overcrowding, lack of basic public services, marginalisation index; (3) characteristics at the local and regional level: locality type and region.

The variables included in the model sought to comply with the balance property for comparison groups, early in 2012 and then in 2016. The 2012 propensity score was obtained through a probit regression, and the resulting equation was used to calculate the 2016 propensity score. Each variable included in the model was tested by linear regression to ensure maximum comparability between beneficiary and non-beneficiary groups.

Propensity score weights were generated by the method described by Stuart *et al.* in 2015^(36)^. For each household (i: 1,…n) group (g: g = 0,1) and time (t: *t* = 0,1), we calculated a propensity score 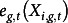 as a function of the covariables 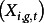 as previously described. Each group (the 2012 comparison group and the 2016 beneficiary and comparison groups) was weighted in an effort to approximate the 2012 beneficiary group. Weights were estimated as follows:
(1)

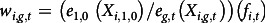




where *w* is weight per household (*i*) in group (*g*) and observation time (*t*) which is proportional to the probability 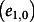 of belonging to 2012 (*t* = 0) beneficiary group (*g* = 1), relative to the probability of their corresponding group and time 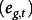 . Furthermore, each was weighted by the expansion factor corresponding to each survey design 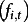 .

Afterwards, a distribution test was performed on models before and after propensity score weighting in order to verify the effect of the weighting on improving the balance between variables. To estimate the effects of PROSPERA, we weighted the DD model with the propensity score 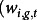 ), considering the expansion factor as shown in equation (1), and using logistic regression according to the model in equation (2):
(2)

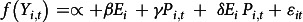

where 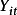 represents FI severity level in a household (i) at a given time (t), and where food security = 0 and FI = 1; 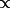 represents FI severity level of the non-beneficiary group (g = 0) at baseline (*t* = 2012), 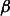 represents differences in FI severity level between 2012 and 2016, 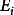 represents the year (2012 or 2016), 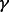 indicates the difference between beneficiaries and non-beneficiaries in 2012, 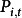 indicates whether a household is beneficiary or non-beneficiary in a given year, 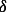 estimates the program effect on FI, 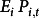 is the interaction term between time (2012 or 2016) and program affiliation (beneficiary or non-beneficiary). Marginal effects were graphed.

Subsequently, we carried out the same analysis while stratifying households by the presence of children, in order to reveal whether or not the effect of the program on FI severity was different by household composition. As a sensitivity analysis, we estimated the DD model weighted only with the complex survey design, as well as the DD model weighted only with the propensity score for all households, and for households with and without children. To analyse the data, we used the complex survey module (SVY) of Stata software, version 14.0.

## Results

We analysed the ENIGH-MCS 2012 and 2016 with data between 56 889 and 70 267 households, respectively. The prevalence of FI in Mexican households between 2012 and 2016 was marked by an increase of food security (2·5pp) as well as of mild FI (0·1pp), in contrast to a decrease in moderate FI (1·3pp) and SFI (1·3pp) (for all, *P*-value > 0·05). Results of FI prevalence are shown in Table 1.

**Table 1:** Population characteristics from the socioeconomic conditions module of the national household income and expenditure survey (ENIGH-MCS) for 2012 and 2016

	ENIGH-MCS 2012		ENIGH-MCS 2016	
*n* (sample)	56 888		70 263	
*n* (total population represented)	31 206 819		33 445 353	
Severity level of household food insecurity	%	95 % CI	%	95 % CI
Food security	59·4	58·6, 60·1	61·9	61·2, 62·5
Mild food insecurity	19·2	18·6, 19·8	19·3	18·9, 19·8
Moderate food insecurity	12·1	11·6, 12·5	10·8	10·5, 11·2
Severe food insecurity	9·3	9, 9·7	8·0	7·7, 8·3
Household member characteristics				
Male head-of-household	74·7	74·1, 75·2	72·3	71·7, 72·8
Education level of head-of-household				
None or kindergarten	8·0	7·7, 8·4	7·3	7, 7·6
Elementary school	36·2	35·5, 36·8	32·5	31·9, 33·1
Middle school	27·5	26·9, 28·1	29·2	28·6, 29·7
High school or more	28·3	27·7, 29	31	30·4, 31·7
Indigenous background	7·5	6·7, 8·2	7·3	6·7, 8·0
Household characteristics				
Quality housing materials				
Lack of flooring materials	3·3	3, 3·6	3·1	2·8, 3·4
Lack of ceiling materials	1·9	1·6, 2·1	1·2	1, 1·3
Lack of wall materials	1·6	1·4, 1·8	1·6	1·4, 1·8
Overcrowding	6·7	6·3, 7	5·6	5·3, 5·9
Basic public services				
Lack of water	8·3	7·5, 9·2	7·2	6·6, 7·9
Lack of sewage	8·4	7·8, 9·1	6·4	6, 6·8
Lack of electricity	0·7	0·6, 0·9	0·5	0·4, 0·6
Lack of gas	11·7	11·1, 12·3	10·6	10·1, 11·1
Marginalisation index				
No marginalisation	27·5	26·9, 28·2	31·8	31·1, 32·4
One dimension	25·5	24·9, 26·1	27·1	26·5, 27·6
Two dimensions	23·5	22·9, 24·1	22·8	22·3, 23·2
Three dimensions	13·6	13·2, 14·1	11·9	11·6, 12·3
Four dimensions	6·8	6·4, 7·1	4·9	4·7, 5·2
Five or six dimensions	3·1	2·8, 3·3	1·6	1·5, 1·7
Local and Regional Characteristics				
Locality type – rural	21·9	21·4, 22·4	21·7	21·3, 22·1
Region				
North	27·2	26·8, 27·6	26·8	26·5, 27·2
Center	35·6	35·1, 36·1	36·2	35·6, 36·9
Mexico City	8·4	8·2, 8·6	8·2	7·9, 8·4
South	28·8	28·4, 29·3	28·8	28·3, 29·3
	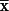	IC95 %	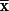	IC95 %
Household size	3·4	3·3, 3·4	3·3	3·3, 3·3
Proportion of women	51·1	50·8, 51·4	51·6	51·3, 51·8
Age of head-of-household	48·7	48·4, 48·9	49·2	49, 49·4

Regarding the characteristics of the study population, heads-of-household were predominantly male across both years. Average age of the head-of-household was 48·7 years in 2012, while in 2016 it was 49·2. Notably, the average education level of heads-of-household in 2012 was lower than in 2016, whereas in both years the average household size was less than 3·5 members per household, and about half of household members were female.

Between 2012 and 2016, the proportion of unfavourable household characteristics such as lack of quality housing materials and basic public services decreased in most dimensions. The proportion of population living in rural localities was similar across both years.

In terms of population distribution across the four regions of Mexico, during both years, more than one-third of the total population resided in the central zone.

Regarding the marginalisation index, during both years, half of the population reported either one or two dimensions of marginalisation.

Supplementary Table shows the variables used to calculate the propensity score. The first column shows that the difference in characteristics between beneficiaries and non-beneficiaries was statistically significant, except for average age of head-of-household (NS before or after). Once the weighting was completed, the differences between variables were no longer statistically significant, such that the groups were considered balanced and comparable. Supplementary Figures show the distribution of the 2012 groups before and after weighting; an improvement was observed in the distribution of the groups at time point 0, revealing similar probabilities of being eligible for PROSPERA.

**Fig. 2:**
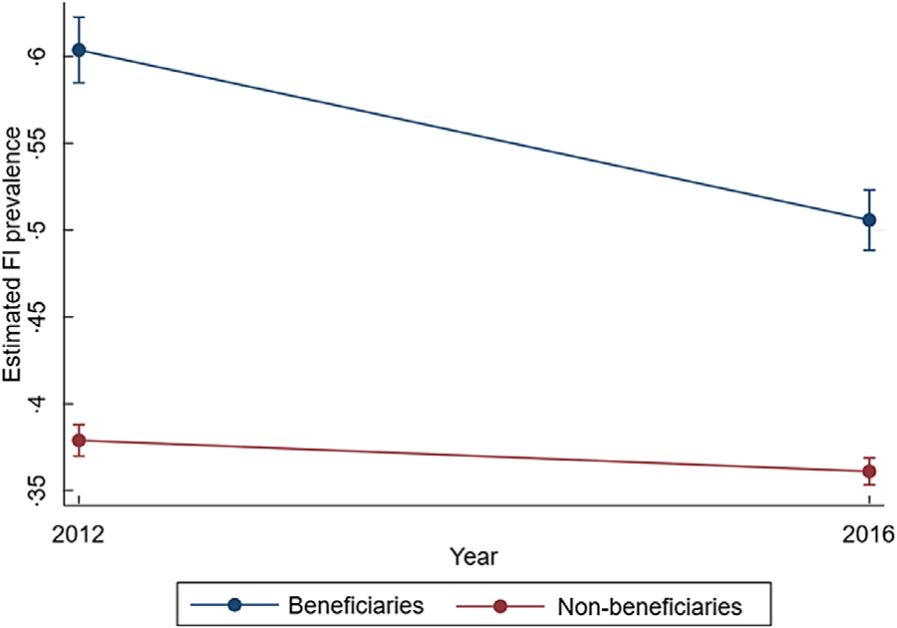
Program effects on food insecurity (FI) in Mexican households from 2012 to 2016

In the DD model, it was found that in both beneficiary and non-beneficiary groups (for all households), there was a decrease in the percentage points on FI between 2012 and 2016. The decrease in FI attributable to being a PROSPERA beneficiary was 8·0pp (95 % CI: 5·3, 10·7), as compared to the non-beneficiary group (Fig. 2).

For households with children, estimated through the DD model with propensity score weighting, we found a decrease in FI severity level within 2 years of 6·0pp (95 % CI 3·0, 9·1), while households without children showed a greater decrease of 12·9pp (95 % CI 8·3, 17·5).

For the model with no propensity score weighting, we observed a decrease in FI severity level for all households of 8·6pp (95 % CI 6·3,11·0) and in households with children, this was 7·4pp (95 % CI 4·7,10·0) and in households without children, this was 10·5pp (95 % CI 6·5,10·5) (for all, *P*-value < 0·001). These results are described in detail in Table 2.

**Table 2:** Sensitivity analysis of the effects of the PROSPERA program on food insecurity. Comparation of results between differences in differences models with and without propensity score weighting

Differences in differences model	Propensity score weighting		*P*-value	No propensity score weighting		*P*-value
	%	IC95 %		%	CI95 %	
All households	–8·0	–10·7, –5·3	<0·001	–8·6	–11·0, –6·3	<0·001
Households with children (<18 years of age)	–6·0	–9·1, –3·0	<0·001	–7·4	–10·0, –4·7	<0·001
Households without children (<18 years of age)	–12·9	–17·5, –8·3	<0·001	–10·5	–14·6, –6·5	<0·001

## Discussion

We studied the effect of PROSPERA, a CCT program to reduce FI in Mexican households, between the years 2012 and 2016. Estimations were performed by propensity score weighting and the DD methodology, and using nationally representative data. Our results showed a decrease in FI severity in Mexican households which may be attributable to PROSPERA; this tendency was true for all households, households with children and households without children over the same time period.

Similar to our findings, Ruiz-Arranz *et al.* analysed PROGRESA household data and observed an increase in total food consumption^(37,38)^ and caloric intake through food purchases in the rural Mexican households studied^(38)^. Results from Angelucci *et al.* are also consistent with these findings, reporting that beneficiary households for Oportunidades consumed more food than they did prior to program affiliation, as estimated using data from the same population^(39)^. In both studies, the reported increase in food consumption and caloric intake could have led to an improvement in FI severity among households. In addition, Mundo-Rosas *et al*. analysed the association between PROSPERA and FI with data from the ENSANUT with less than 100 000 inhabitants (ENSANUT 100K), reporting that beneficiary households were less likely to report FI (OR 0·72; 95 % CI 0·51, 1·02) as compared to non-beneficiary households^(40)^.

These results have also been consistent throughout Latin America. The cash transfer programs ‘Familias en Acción (Families in Action) in Colombia and ‘Bolsa Família’ (Family Fund) in Brazil are CCT which provide households with a higher income, and which have demonstrated outcomes of improved food access^(41,42)^.

Although CCT programs improve food access in countries like Brazil, it has been observed that the degree of dependence on CCT income is positively associated with an increased self-reported intake of sugar and soft drinks^(43)^, demonstrating that greater food access does not equate to higher diet quality. However, from its conception, the PROSPERA program has included nutritional health counselling^(44)^ in order to encourage families to make wise food choices^(38)^.

Miller *et al.* studied the Social Cash Transfer Scheme, and reported a positive impact of cash transfers on food security in rural Malawi through the provision of income necessary for households to increase food expenditures as well as share of expenditures dedicated to food^(45)^. Although transfers have been affirmed as improving food security, program implementation can define whether or not the program’s desired FI outcomes are achieved. This was demonstrated in a multi-country analysis in sub-Saharan Africa in which findings indicated that predictable and regular cash transfers (such as PROSPERA) allow beneficiary households to plan and distribute food consumption over the full time period until the next payment, whereas sporadic and irregular payments produced less reports of significant effects on food security^(46)^.

In our study, we observed that a greater decrease (12·9pp) was achieved in FI for households without children, whereas in households with children this FI decrease was cut into half (6·0pp). This may be because in households without children, the income from the cash transfer provides security to the family and the perception of FI decreases in greater proportion as compared to households with children. Furthermore, in a study of rural Mexico, households who were beneficiaries of PROSPERA and PROCAMPO (a program aimed to support farmers) registered an improved diversity in foods consumed, replacing quantity for quality by turning from high caloric density foods to others such as meat and vegetables^(38)^. In households with children, when FI with moderate hunger exists, adults may skip meals or reduce their own portions in order to provide for their children^(47)^. Thus, according to the EMSA, households just beginning to experience FI first decrease the variety of foods consumed (quality), and only subsequently decrease quantity^(32)^.

In a previous study in Mexico, Valencia *et al*. reported that FI is more prevalent in households with children than those without children. Furthermore, the most severe levels of FI occur when children are affected^(4)^. We can therefore expect that mitigating the FI of a household with children is more difficult than doing so in households without children, given the more severe FI of the prior.

This study has certain limitations. Ideally, program effects should be estimated using an experimental design; however, for ethical reasons, the randomised allocation of beneficiaries was not permitted in PROSPERA’s design, making it difficult to validate comparisons between groups. It should be noted that the treatment groups for 2012 and 2016 were not panels; nevertheless, propensity weight scoring within the statistical analysis allowed for comparability between groups.

Comparing our reported results against the DD models with no propensity score weighting, we observed that the estimations of FI changes for all households and for households with children were overestimated, whereas for households without children, the model underestimated the decrease in FI. These differences may be explained by the use of the two comparison groups over two time points, or by propensity score weighting groups in the DD models used to control confusion attributable to observed variables that differed either between groups at time point 0, or over time due to changes in group composition. The methodology applied balances each group so they are similar to the 2012 beneficiary group (at time point 0)^(36)^. Therefore, the degree of balance between groups was measured to ensure comparability.

There have been changes to this CCT program since it began in 1997. In this study, we estimated the effect of the changes made to the program on FI in 2016, however, any change to the program in 2014 may have modified its effect on FI, although this was not possible to determine in this study.

Regarding the potential role of the program activities on the demonstrated reduction of FI in households conformed by only adults, as well as households with children, according to the program’s operational procedures,^(48)^ it provides four types of food support through additional cash transfers, in addition to food supplements for mothers and young children. Of these, three are targeted to households, and one of them specifically to children from 0 to 9 years of age. Since most transfers are targeted to households, it is possible that FI could have been reduced not only in households with children, but also in households without.

The information analysed was from a secondary database, although it was obtained from the National Institute of Statistics and Geography (INEGI) which carries out the ENIGH. Therefore, the information is of national interest and highly relevant for public policy planning.

Our study adds greater evidence describing the positive effects of PROSPERA on beneficiary households. Our findings may contribute to policy decisions related to population nutrition, since it demonstrates further positive effects of the program, adding to those previously documented in child, women and pregnant women nutrition.

PROSPERA program was terminated in 2019, understanding its effect on FI provides greater arguments for the improvement of the social rights of the population. Indeed, the findings of Palmeira *et al.* suggest that low or null investment in cash transfer programs directly determines the persistence of FI among vulnerable populations^(49)^. The vulnerability of our study population was confirmed by the results of the marginalisation index, which assumes equal relevance for all dimensions of social access such that the lack of one dimension affects the fulfillment of the others. Only one-third of the study population reported all six dimensions being satisfied; the rest of the population is on the threshold of marginalisation (lacking one dimension) or worse, which implies significant social disadvantages^(33)^. Due to the high prevalence of FI in the Mexican population, it is critical to immediately incorporate policies proven to be effective in reducing and preventing FI in vulnerable households, especially in those with children under 5 years of age.

## Conclusions

The PROSPERA program had a positive effect on decreasing FI at the household level, by way of the cash transfers which allowed increased food access to households. This could improve nutritional outcomes in vulnerable groups within the Mexican population. Understanding the effects of the program provides evidence for reestablishing this type of approach using an even broader focus, in order to protect the social rights of the population. The elimination of PROSPERA without a replacement may increase the risk of FI and its consequences in the population, especially in vulnerable households and for children. Without it, families have lost the regularity of the cash transfers which previously provided them with a stable income source (an important component for enabling food access), and potentially also the healthcare services stipulated as a transfer condition, potentially leading to health complications or illness.

## Supplementary material

For supplementary material accompanying this paper visit https://doi.org/10.1017/S1368980021003918. click here to view supplementary material
