# Supplementary material for: Effect of a conditional cash transference program on food insecurity in Mexican households: 2012–2016
Source: Public Health Nutr. 2021 Sep 9;25(4):1084–93. doi: 10.1017/S1368980021003918 (PMC9991821; doi:10.1017/S1368980021003918)
Supplement: Supplementary file 1 [file S1368980021003918sup.zip › S1368980021003918sup002.pdf]

Supplementary Table. Demographic characteristics of beneficiaries and non-beneficiaries in 2012, before and after propensity score weighting.

**Propensity Score Variables**	**Before Weighting**			**After Weighting**		
	Beneficiaries	Non-Beneficiaries	*p*-value	Beneficiaries	Non-Beneficiaries	*p*-value
	% (IC95%)	% (IC95%)		% (IC95%)	% (IC95%)	
**Household Member Characteristics**						
Sex of head-of-household	77.1(76.3,77.9)	74.1(73.7,74.5)	<0.001	75.6(73,78.1)	75.0(74.5,75.6)	0.654
Education level of head-of-household			<0.001			0.505
None or Kindergarten	18(17.3,18.7)	6.3(6.1,6.5)		8.9(8.2,9.7)	9.3(8.2,10.5)	
Elementary school	54.1(53.1,55)	33.9(33.5,34.3)		36.9(34.4,39.4)	37.6(36.9,38.3)	
Middle school	22.3(21.5,23.1)	28.2(27.8,28.6)		26.7(24.5,29.1)	26.8(26.2,27.3)	
High school or more	5.7(5.2,6.1)	31.6(31.1,32)		27.5(23.5,31.9)	26.3(25.8,26.9)	
Indigenous background	23.2(22.4,24)	5.5(5.3,5.7)	<0.001	9.4(8.5,10.4)	9.2(8.7,9.8)	0.724
Household size	4.1(4,4.1)	3.2(3.2,3.2)	<0.001	3.6(3.5,3.7)	3.5(3.3,3.7)	0.222
Proportion of women	52.2(51.8,52.6)	50.6(50.4,50.9)	<0.001	51.0(49.9,52.1)	50.9(50.7,51.2)	0.958
Average age by tertile			0.514			0.452
Tertile 1	33.6(33.4,33.7)	32.7(32.3,33.2)		31.7(31.6,31.8)	31.7(31.6,31.8)	
Tertile 2	47.3(47.1,47.4)	46.7(46.4,47.1)		47.9(47.8,48.0)	48.0(47.9,48.1)	
Tertile 3	68(67.7,68.3)	67.6(66.2,69)		67.5(67.4,67.7)	67.7(67.5,67.8)	
**Household Characteristics**						
Lack of healthcare access	13.2(12.6,13.9)	18.8(18.5,19.2)	<0.001	16.8(15,18.7)	17.6(17.2,18.1)	0.381
Economic vulnerability	1.6(1.4,1.8)	6.2(5.9,6.4)	<0.001	5.1(4.2,6.2)	5.2(5,5.4)	0.802
Quality housing materials						

Lack of flooring materials	8.3(7.8,8.8)	2.3(2.2,2.4)	<0.001	3.9(3.5,4.4)	3.8(3.5,4.2)	0.648
Lack of ceiling materials	3.9(3.5,4.3)	1.3(1.2,1.4)	<0.001	1.8(1.6,2.1)	1.9(1.6,2.1)	0.753
Lack of wall materials	4.5(4.1,4.9)	1.2(1.1,1.3)	<0.001	2.0(1.7,2.3)	1.9(1.7,2.1)	0.515
Overcrowding	15.9(15.2,16.6)	4.7(4.5,4.9)	<0.001	8.1(7.4,8.8)	8.1(7,9.3)	0.995
Basic public services						
Lack of water	18.4(17.7,19.2)	5.4(5.2,5.6)	<0.001	8.0(7.4,8.7)	8.0(7.6,8.4)	0.954
Lack of sewage	24.8(24,25.6)	5.2(5,5.5)	<0.001	9.3(8.6,10.1)	9.3(8.8,9.9)	0.936
Lack of electricity	1.7(1.5,2)	0.7(0.6,0.7)	<0.001	1.0(0.8,1.2)	0.9(0.8,1.1)	0.823
Lack of gas	41.1(40.2,42.1)	7.3(7.1,7.6)	<0.001	14.5(13.5,15.6)	14.2(13.6,14.8)	0.593
Marginalization index			<0.001			0.482
No marginalization	3.5(3.1,3.8)	31.9(31.5,32.4)		28.6(24.6,33)	26.2(25.7,26.7)	
One dimension	16.1(15.4,16.8)	27.9(27.5,28.3)		24.1(22.1,26.2)	25.3(24.8,25.8)	
Two dimensions	26.9(26.1,27.8)	22.6(22.2,23)		21.6(20,23.4)	23(22.5,23.5)	
Three dimensions	27.5(26.7,28.4)	10.8(10.5,11.1)		14.5(13.3,15.8)	14.5(13.4,15.6)	
Four dimensions	17.8(17.1,18.5)	4.7(4.6,4.9)		7.6(7,8.3)	7.4(7,7.9)	
Five or six dimensions	8.2(7.7,8.7)	3.6(3.2,4.1)		3.5(3.2.3.9)	3.6(3.2,4.1)	
**Local and Regional Characteristics**						
Locality type			<0.001			0.796
Urban	39.3(38.4,40.2)	82.8(82.5,83.2)		73.2(71.4,75)	73.5(72.4,74.6)	
Rural	60.7(59.8,61.6)	17.2(16.8,17.5)		26.8(25,28.6)	26.5(25.4,27.6)	
Region			<0.001			0.140
North	22.9(22.1,23.7)	36.2(35.8,36.6)		34.7(31.8,37.8)	33.2(32.6,33.8)	
Center	25.2(24.4,26.1)	33.0(32.6,33.5)		31.5(28.4,34.7)	31.4(30.8,32)	

Mexico City	0.2(0.2,0.4)	3.9(3.7,4.1)	3.3(1.4,7.5)	3.1(3,3.3)
South	51.6(50.7,52.6)	26.9(26.5,27.3)	30.5(28.3,32.8)	32.3(31.3,33.3)
