# Supplementary material for: Effect of a conditional cash transference program on food insecurity in Mexican households: 2012–2016
Source: Public Health Nutr. 2021 Sep 9;25(4):1084–93. doi: 10.1017/S1368980021003918 (PMC9991821; doi:10.1017/S1368980021003918)
Supplement: Supplementary file 1 [file S1368980021003918sup.zip › S1368980021003918sup003.pdf]

Supplementary Material. EMSA Questionnaire

The EMSA questionnaire is described below. Every question begins with the phrase "Due to lack of money or resources…" followed by: 1. Have you or any adult in your household ever had a diet with too little food variety?; 2. Have you or any adult in your household ever skipped breakfast, lunch or dinner?; 3. Have you or any adult in your household ever ate less than you thought you should?; 4. Have you or any adult in your household ever run out of food?; 5. Have you or any adult in your household ever felt hungry but did not eat?; 6. Have you or any adult in your household ever only eaten once in a day, or not eaten for a whole day?; 7. Has any child under 18 in your household ever eaten a diet with too little food variety?; 8. Has any child under 18 in your household ever eaten less than they should have?; 9. Have you ever had to reduce the food portions served to any child under 18 in your household?; 10. Has any child under 18 in your household ever been hungry but did not eat?; 11. Has any child under 18 in your household ever gone to bed hungry?; 12. Has any child under 18 in your household ever eaten only once in a day, or not eaten for a whole day?[(32)]
