# Supplementary figures and images for: Effect of a conditional cash transference program on food insecurity in Mexican households: 2012–2016
Source: Public Health Nutr. 2021 Sep 9;25(4):1084–93. doi: 10.1017/S1368980021003918 (PMC9991821; doi:10.1017/S1368980021003918)

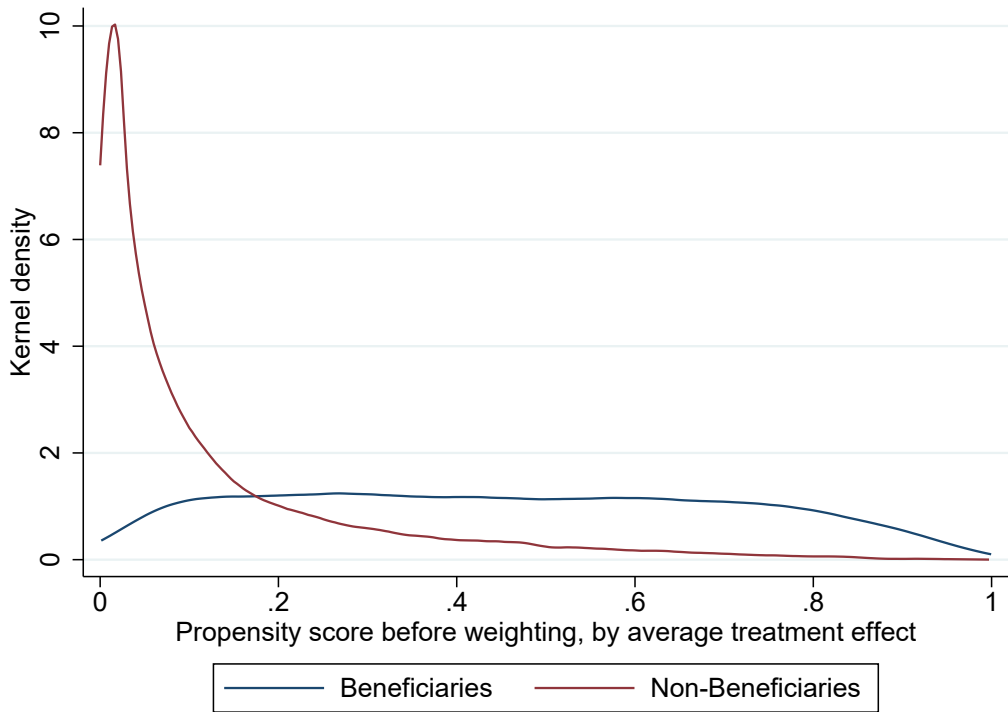

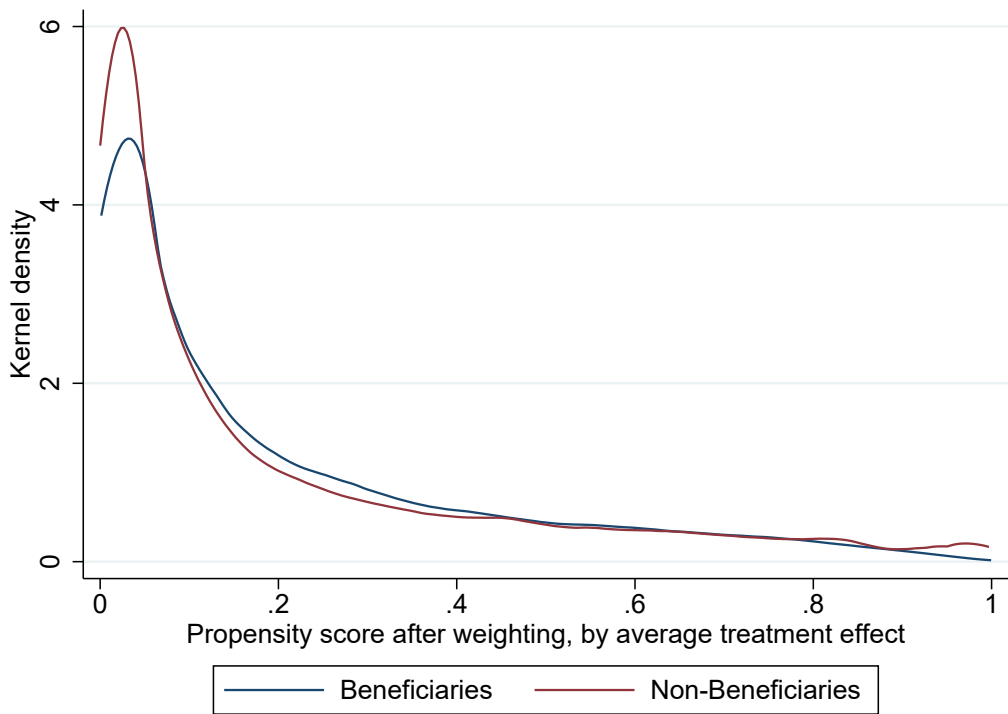

Supplement: Supplementary file 1 [file S1368980021003918sup.zip › S1368980021003918sup001.pdf]
